# Histoepigenetic analysis of the mesothelin network within pancreatic ductal adenocarcinoma cells reveals regulation of retinoic acid receptor gamma and AKT by mesothelin

**DOI:** 10.1038/s41389-020-00245-3

**Published:** 2020-07-02

**Authors:** Eugene Lurie, Dongliang Liu, Emily L. LaPlante, Lillian R. Thistlethwaite, Qizhi Yao, Aleksandar Milosavljevic

**Affiliations:** 1 Department of Molecular and Human Genetics, Baylor College of Medicine, Houston, TX USA; 2 Michael E. DeBakey Department of Surgery, Baylor College of Medicine, Houston, TX USA; 3 Program in Quantitative and Computational Biosciences, Baylor College of Medicine, Houston, TX USA; 4 Center for Translational Research on Inflammatory Diseases (CTRID), Michael E. DeBakey VA Medical Center, Houston, TX USA; 5 Present Address: Department of Translational Molecular Pathology, The University of Texas M. D. Anderson Cancer Center, Houston, TX USA

**Keywords:** Pancreatic cancer, Tumour heterogeneity, Cancer genomics

## Abstract

To enable computational analysis of regulatory networks within the cancer cell in its natural tumor microenvironment, we develop a two-stage histoepigenetic analysis method. The first stage involves iterative computational deconvolution to estimate sample-specific cancer-cell intrinsic expression of a gene of interest. The second stage places the gene within a network module. We validate the method in simulation experiments, show improved performance relative to differential expression analysis from bulk samples, and apply it to illuminate the role of the mesothelin (MSLN) network in pancreatic ductal adenocarcinoma (PDAC). The network analysis and subsequent experimental validation in a panel of PDAC cell lines suggests AKT activation by MSLN through two known activators, retinoic acid receptor gamma (RARG) and tyrosine kinase non receptor 2 (TNK2). Taken together, these results demonstrate the potential of histoepigenetic analysis to reveal cancer-cell specific molecular interactions directly from patient tumor profiles.

## Introduction

The study of oncogene expression in unperturbed cancer cells within their tumor microenvironment provides insights that may not be gained from other more accessible models that may not reflect as closely the tumor biology in vivo*.* The lack of correspondence of experimental models with tumor biology in vivo may lead to only partial understanding of oncogenic mechanisms, ultimately resulting in failures of experimental therapies targeting the oncogene. One illustrative example is the glycosylphosphatidylinositol-anchored cell-surface protein mesothelin (MSLN), whose expression is elevated in many pancreatic ductal adenocarcinoma (PDAC) cases^1^ and is associated with shorter overall survival^2^. Most of the current knowledge about MSLN’s function in the context of PDAC comes from mouse or cell-line models, which have shown its upregulation increases cancer cell proliferation, cell cycle progression, and cell survival^3–5^; while its interaction with MUC16 contributes to cell motility and invasion^6,7^. These models have shown limited utility for developing therapies targeting MSLN: while preclinical studies targeting MSLN in pancreatic cancer cell lines and patient-derived xenograft mouse models have resulted in inhibition of cancer growth^8,9^, early-stage clinical trials have shown stabilized disease or increase in survival for only a few patients^10,11^, with partial and overall responses being mostly low or absent^12,13^.

One obvious step toward addressing this problem is to study the oncogene and its network within its natural tumor microenvironment. However, transcriptional profiling of tumors is confounded by sample-to-sample differences in tumor purity and cell-type composition^14,15^. This is a particularly important concern for PDAC tumors as they can show an abundant and highly variable stromal component, leading to ambiguous interpretation of bulk expression profiles^16^. For example, a sample with higher bulk expression of *MSLN* may either contain cancer cells with higher intrinsic expression of *MSLN* or the expression of *MSLN* may be constant and the sample may just have higher purity (proportion of cancer cells), resulting in higher bulk expression of the gene. This ambiguity hampers not only cancer-cell intrinsic differential gene expression analysis, but also the analysis of genes of interest in the context of their cancer-cell-specific regulatory networks.

One approach toward addressing this problem would be to computationally tease apart the associated cancer cell-type-specific differences between groups of tumors. Numerous computational methods have been developed to account for tumor purity differences^14^, but cannot explicitly trace differential expression to specific cell types nor place the gene within its network module. Our previously developed Epigenomic Deconvolution (EDec) method^17^ partially addresses this problem by comparing cell-type-specific profiles starting from two groups of predefined groups of samples. One obvious limitation of EDec is the requirement that the two groups be predefined. This limitation precludes grouping by cell-intrinsic expression of genes of interest such as *MSLN*. To address this “chicken and egg” problem, we develop a histoepigenetic analysis method that expands on the EDec method in two ways: (1) It integrates within an iterative procedure a two-way clustering step with deconvolution to separate PDAC tumors based on cancer-cell-specific expression of a gene of interest such as *MSLN*; (2) It estimates a network of genes potentially interacting with the gene of interest. We here validate the method in simulation experiments, compare it to alternative approaches, and apply it to elucidate MSLN’s network within PDAC cancer cells.

## Results

### Deconvolution of TCGA PDAC datasets identifies four constituent cell types

**Fig. 1:**
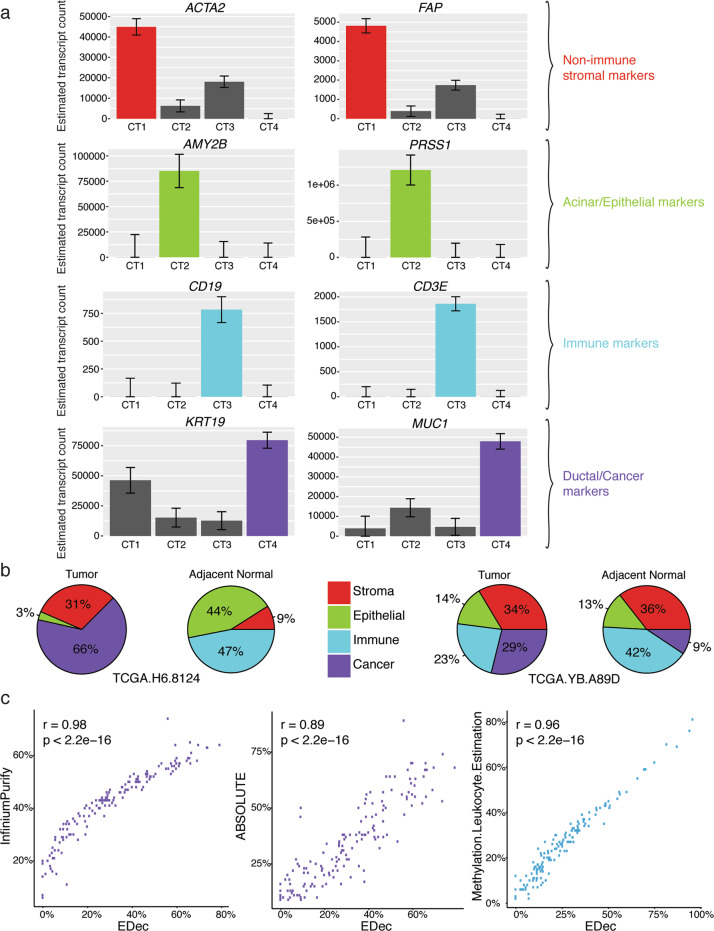
EDec deconvolution of TCGA PDAC dataset. **a** Bar plots showing assignment of mean expression, with standard error, of 8 marker genes to the 4 predicted cell types (CT) from the gene expression deconvolution of 150 TCGA samples. Colors represent different constituent cell types. **b** Pie charts showing estimated composition of tumor samples and paired adjacent normal samples for two patients from the TCGA collection. **c** Scatter plots showing Spearman’s correlations between EDec estimates of cancer cell proportions and previously generated tumor purity estimates by InfiniumPurify (left) and ABSOLUTE (center) and EDec estimates of immune cell proportions with those also previously generated (right).

We first applied the original EDec method to the DNA methylation and gene expression profiles of 150 PDAC tumors and 2 adjacent normal tissues within The Cancer Genome Atlas (TCGA) collection. EDec estimated four distinct cell type profiles, provided their per sample proportions, and provided estimates of cell-type-specific DNA methylation and expression profiles. Based on expression of known cell-type-specific marker genes, the profiles could be unambiguously recognized as belonging to stromal, immune, normal epithelial/acinar, and cancer cell types (Fig. 1a). Comparisons of deconvoluted tumor tissue to deconvoluted paired adjacent normal tissue showed a larger proportion of the predicted cancer cell type in the tumors compared to the normal tissues (Fig. 1b). The predicted cancer cell proportion in the normal tissue of patient TCGA.YB.A89D is consistent with previous central pathology review that noted “~10% neoplastic cellularity”^15^. Per sample estimates of cancer cell proportions for these 150 tumors showed high Spearman’s correlation with previous tumor purity estimates calculated using InfiniumPurify^18^ (*r* = 0.98, *p* < 2.2 × 10−16) and ABSOLUTE^15^ (*r* = 0.89, *p* < 2.2 × 10−16) (Fig. 1c). Likewise, precalculated leukocyte proportion estimates^15^ showed high correlation with immune cell proportion estimates (*r* = 0.96, *p* < 2.2 × 10−16) (Fig. 1c). These results suggest EDec properly estimated constituent cell types, their proportions within individual samples, and cell-type-specific expression profiles.

### Grouping tumor samples by cancer-cell intrinsic expression of *MSLN*

**Fig. 2:**
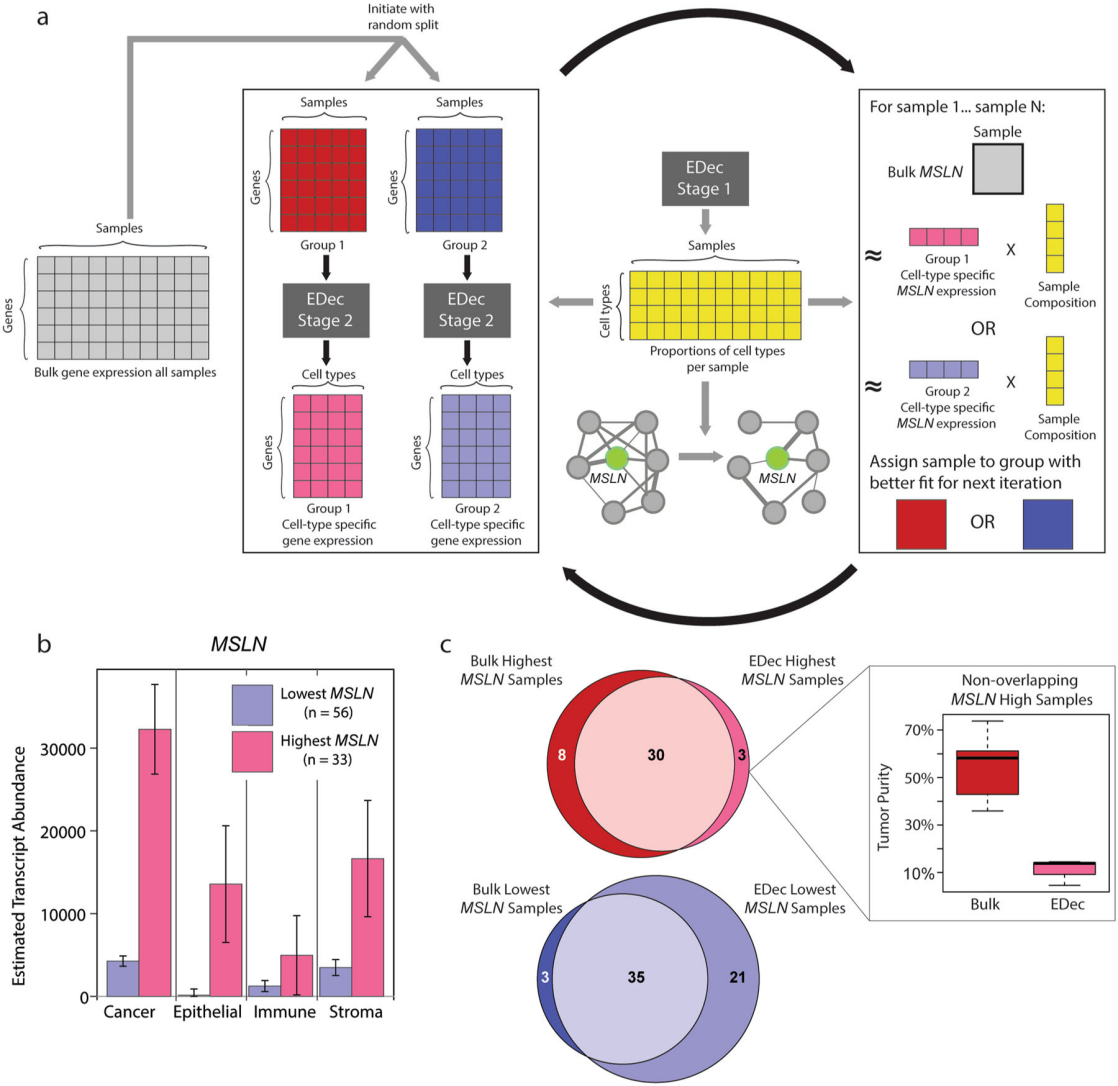
Histoepigenetic analysis method. **a** EDec stage 1 identifies constituent cell types and produces a matrix (yellow, center) of proportions of cell types per samples. In the first stage of histoepigenetic analysis, the matrix is used for iterative computational deconvolution to estimate sample-specific cancer-cell intrinsic expression of a gene of interest (arrows from the yellow matrix to the left and right). In the second stage, a network is constructed and pruned to eliminate confounding due to variation in cell-type composition (arrow from the yellow matrix down) to place the gene within a network module. **b** Bar plot of mean cell-type specific *MSLN* expression within “*MSLN* high” and “*MSLN* low” tumors identified by histoepigenetic analysis. **c** Comparison of “*MSLN* high” vs “*MSLN* low” tumor classification from bulk profiles vs. histoepigenetic analysis. Euler diagrams show overlap of samples grouped into the first (red) and third (blue) quartiles by the two methods. The callout shows boxplots of tumor purity for the 8 non-overlapping samples. Note that the samples classified as having high *MSLN* based on bulk levels (red) show much higher purity (cancer cell proportion) than the 3 non-overlapping samples classified as high *MSLN* based on histoepigenetic analysis but not by bulk profile comparison.

The goal at the first stage of histoepigenetic analysis (see “Materials and methods” and Fig. 2a) was to group tumor samples based on cancer cell intrinsic *MSLN* expression—addressing complications introduced by tumor purity differences (Supplementary Fig. 1)—and to compare those with high vs. low expression. To test the performance of the method, we ran several simulations on mock pancreatic sample profiles containing reference cancer cell profiles with either high or low *MSLN* expression. Simulated mixing showed high correlation between estimated proportions and known mixed proportions (Supplementary Fig. 2a) and stable clustering into *MSLN* high vs *MSLN* low tumors (Supplementary Fig. 2b). Moreover, deconvoluted cell-type-specific gene expression profiles of these groups were also accurate (Supplementary Fig. 2c). Taken together, the results suggest that the method performed accurately. We next applied the method to the PDAC tumors in the TCGA dataset to identify and compare those with high vs. low cancer cell-intrinsic expression of *MSLN*. The first split yielded one group of PDACs with high cancer cell intrinsic *MSLN* expression and the other with lower expression. In the second step, the latter was further split into two groups (Supplementary Fig. 3a). The group with the lowest *MSLN* expression was then compared to the one with the highest (Fig. 2b).

We next compared the result of iterative grouping to grouping based on bulk profiles. For this purpose, we split samples into three quartiles for each method and compared the samples in the top (first) and bottom (third) quartile for each method. While the overlap was high (Fig. 2c), the exceptions could be explained by the confounding effect of tumor purity: bulk grouping incorrectly assigned 8 samples with low cancer cell *MSLN* levels but high tumor purity and missed 3 samples with high cancer cell *MSLN* levels but low tumor purity. Consistent with this finding, no significant differences were observed in the predicted cell-type proportions between the *MSLN* high vs *MSLN* low quartiles generated by the iterative method (Supplementary Fig. 3b), while the quartiles obtained from bulk profiling showed significant (*p* < 0.05, Wilcoxon rank-sum test) differences in several cell-type proportions—notably cancer—between groups (Supplementary Fig. 3c). Moreover, we also observed smaller survival differences between high and low groups based on cancer-cell intrinsic expression of *MSLN* compared to bulk expression levels of *MSLN* (Supplementary Fig. 4a). This observation suggests that survival differences observed from bulk profiling may be due to confounding effects of cell-type composition and tumor size^19^ evident in bulk comparisons (Supplementary Fig. 4b, c).

For the purpose of subsequent analyses, we focused on the sample set showing concordant *MSLN* high expression by both groupings and the sample set showing concordant *MSLN* low expression also by both groupings (30 and 35 samples respectively, indicated within circle overlaps in Fig. 2c).

### Regulation of *RARG* by MSLN

We next examined genes whose cancer-cell intrinsic expression correlates with *MSLN*. Comparing cancer cell profiles between groups, 33 genes showed higher expression in the *MSLN*-high group and 55 showed lower expression (Supplementary Table 1). In addition to *MSLN* itself^20^, several of the 33 genes with higher expression within the *MSLN*-high group of tumors are associated with poor prognosis and tumor progression in PDAC patients, including *KCNN4* (ref.^21^), *TNK2* (ref.^22^), *CAPG*^23^, and *MUC1* (ref.^24^). Gene set enrichment analyses (GSEA) of these 33 genes identified a highly enriched subset of 11 genes bound by retinoic acid receptor gamma (RARγ/ RARG) in mice^25^, including *MSLN* and *RARG* itself (Supplementary Table 2). This enrichment is biologically significant because RARγ is expressed in human pancreatic carcinomas^26^ and plays a role in pancreatic duct differentiation^27^. Notably, *RARG* was one of 22 genes not detected as being differentially expressed by bulk expression comparisons (Supplementary Table 1).

**Fig. 3:**
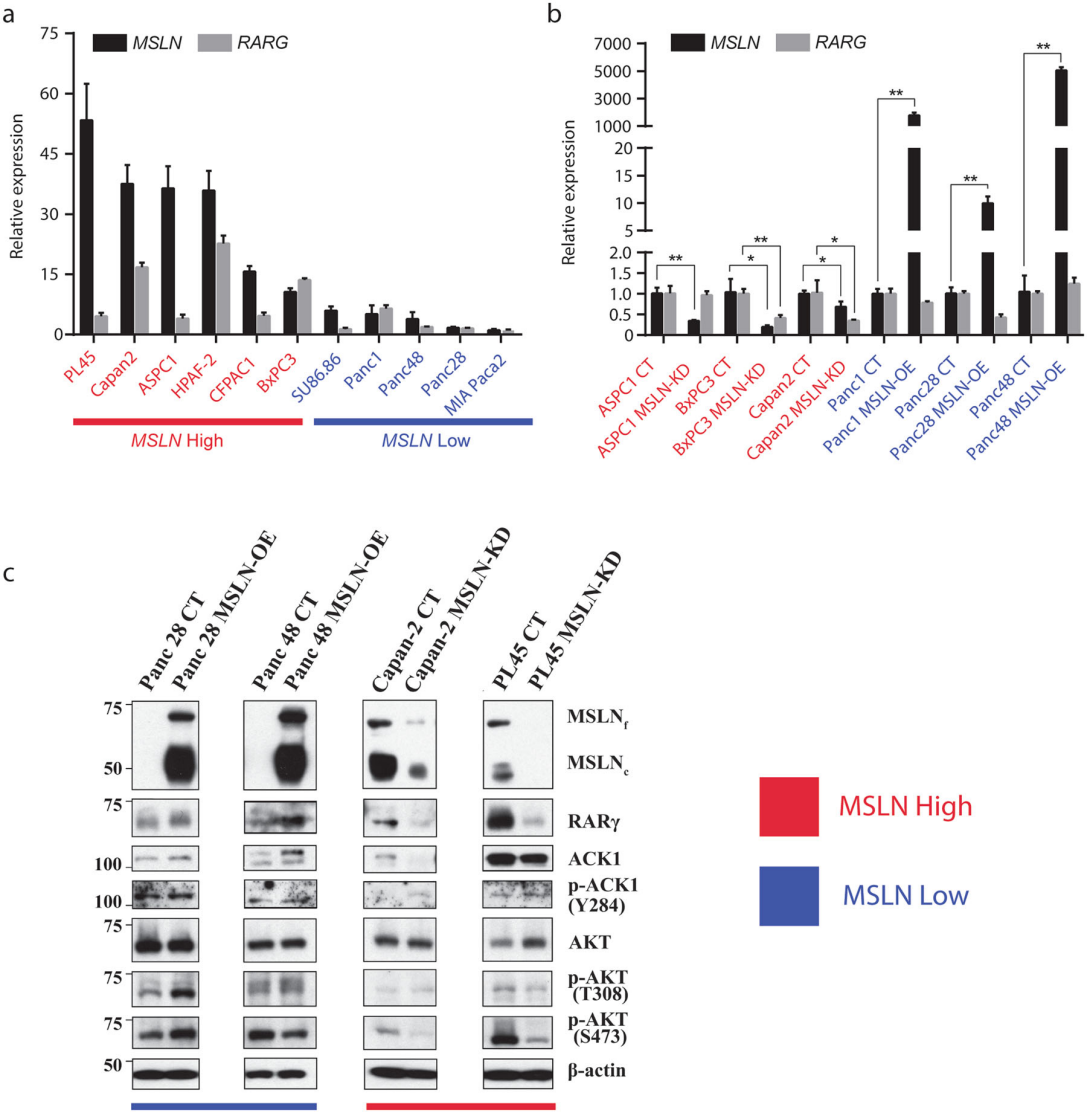
Effects of MSLN modulation on upstream activators of AKT. **a** Bar plots showing real-time PCR results of *MSLN* and *RARG* expression across a panel of PDAC cell lines. Cell lines with high *MSLN* expression are colored red, while the ones with low expression are colored blue. **b** Bar plots showing real-time PCR results of *MSLN* and *RARG* expression in control (CT) *MSLN* high cell lines (red) compared to their counterparts with *MSLN* knocked down (MSLN-KD) and control *MSLN* low cell lines (blue) compared to their counterparts with *MSLN* overexpressed (MSLN-OE). Mean and standard deviations are shown for three independent experiments (*p < 0.05, **p < 0.01). **c** Immunoblot assays for the expression of full length/precursor MSLN (MSLNf), cleaved/mature MSLN (MSLNc), RARγ, total ACK1, phosphorylated ACK1, total AKT, and phosphorylated AKT at two different residues in control (CT) *MSLN* high cell lines (red) compared to their counterparts with *MSLN* knocked down (MSLN-KD) and control *MSLN* low cell lines (blue) compared to their counterparts with *MSLN* overexpressed (MSLN-OE).

To explore the mechanistic link between *MSLN* and *RARG* expression, we utilized a panel of pancreatic cancer cell lines with a range of *MSLN* levels^4^. Cancer cell lines with higher levels of *MSLN* indeed showed a trend toward higher levels of *RARG* (Fig. 3a). Upon *MSLN* knockdown, 2 of 3 cell lines with high *MSLN* levels showed significantly (*p* < 0.05, two-tailed unpaired Student’s *t*-test) reduced *RARG* transcription (Fig. 3b). Conversely, upon overexpression of *MSLN*, the cell lines with low *MSLN* did not show an increase in *RARG* levels (Fig. 3b), suggesting that MSLN may be necessary, but not sufficient for *RARG* expression.

### Activation of the AKT pathway by MSLN

Among the 33 genes in the *MSLN*-high group, RARG and the non-receptor tyrosine kinase TNK2 (or ACK1) stood out for their previously demonstrated ability to phosphorylate and activate AKT. Specifically, ACK1 activates AKT in pancreatic cancer where it also promotes cell survival and correlates with disease progression^22^. On the other hand, RARγ is known to activate AKT in hepatocellular carcinoma^28^. These findings were intriguing in light of the previous results implicating MSLN in the activation of AKT in PDAC cell lines via phosphorylation^5^ by an unknown mechanism.

We next asked if RARγ and ACK1 are the mediators of AKT activation. To answer this question, we knocked down *MSLN* in the cell lines with high *MSLN* expression. As predicted, the knockdown reduced RARγ and ACK1 levels (Fig. 3c), as well as reduced phospho-AKT levels (Fig. 3c**)** at both residues (S473 and T308) necessary for AKT activation^29^. Upon *MSLN* overexpression in cell lines with low *MSLN* expression, protein levels of RARγ and ACK1 were increased (Fig. 3c), consistent with their mediating role in AKT activation. Notably, in contrast to the increase of protein levels of RARγ and ACK1, MSLN overexpression did not alter their transcriptional levels.

We next asked if the downstream effects of MSLN-mediated activation of AKT observed in cell line studies^5^ also occurs in patient tumors. Toward this end, we performed enrichment analysis on the genes that show higher cancer-cell-specific expression in the *MSLN*-high group of tumors. Indeed, a significant (*p* = 0.004, hypergeometric test) overlap was found with genes previously shown to be activated by AKT in vitro, suggesting that the in vitro cell line modeling indeed reflects the behavior of cancer cells in vivo.

### AKT activators RARG and TNK2 belong to the MSLN network module

The goal of the next (second) stage of histoepigenetic analysis (see “Materials and methods” and Fig. 2a) was to directly construct the *MSLN* network module, validate direct interactions of MSLN with *TNK2* and *RARG* within the module, and identify additional genes relevant for understanding MSLN function in cancer cells. Toward this goal, we inferred a Gaussian graphical model (GGM) from the 150 bulk PDAC tumor expression profiles in the TCGA collection (Materials and Methods). To eliminate confounding due to variations in tumor purity, cell-type proportions (inferred by deconvolution) were added as covariates (Materials and Methods). The final GGM included the 367 nodes corresponding to the genes differentially expressed between *MSLN*-high and low tumors within any of the four estimated cell types (Fig. 1) and 6,130 edges.

**Fig. 4:**
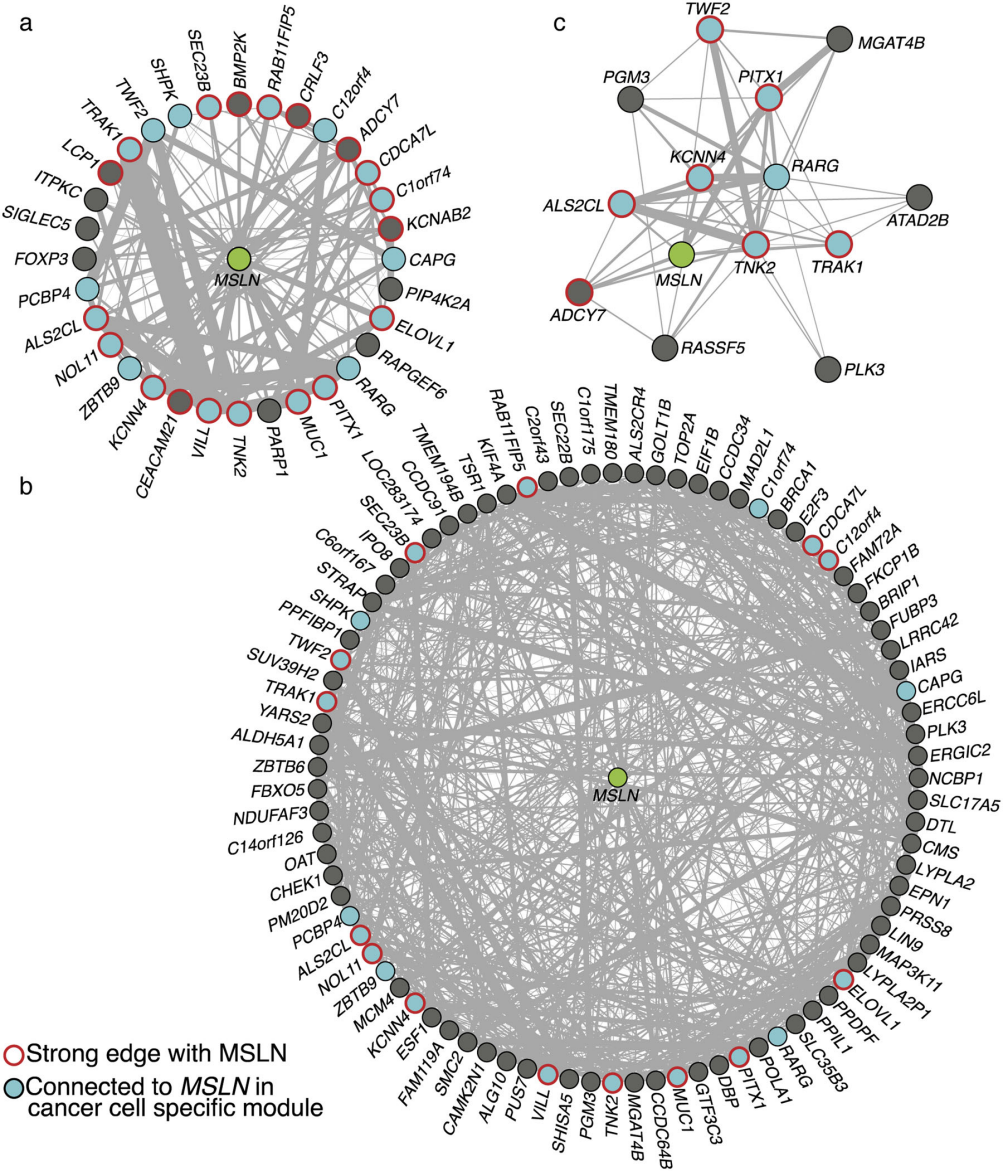
Network connectivity of *MSLN* to genes with differential expression. **a** Subgraph of the GGM network that includes *MSLN* and its first-order neighbors that are differentially expressed in any cell type within PDACs. Line weights are scaled to the absolute value of the regularized partial correlation between genes. **b** Subgraph showing the 88 genes differentially expressed in cancer cells; note that some, but not all of the genes are first-order neighbors of *MSLN*. **c** Subgraph including *RARG*, *TNK2* and all 12 genes (including *MSLN*) that are first-order neighbors of both *RARG* and *TNK2* within the GGM.

We next examined the 32 of 367 genes that were directly connected with *MSLN*. Notably the 32 included *TNK2* and *RARG* (Fig. 4a), validating their direct interactions with *MSLN*. Moreover, GGM also recapitulated known interactions of RARγ, showing a significant (*p* < 1.98×10-12, hypergeometric test) enrichment of connections between *RARG* and genes with known motifs for RARγ^30^ termed retinoic acid response elements (RAREs) (Supplementary Table 3).

We then focused on the 88 genes showing differential cancer cell expression between *MSLN*-high and low groups (33 showing higher expression in the *MSLN*-high group and 55 showing lower expression, Supplementary Table 1). We next applied CTD, an in-house developed method (see “Materials and methods”), to analyze the genes in a network context by figuratively “connecting the dots” in the network. Briefly, CTD takes a set of genes and a weighted graph (the network) and identifies any subsets of the gene set that shows significant pattern of connectedness within the graph together with a *p* value for that pattern. CTD identified significant pattern of GGM connections between some members of this set (*p* < 1.8 × 10–47) (Fig. 4b). Moreover, seven differentially expressed genes identified via GSEA to be bound by RARγ, including *MSLN* and *TNK2*, also formed a significantly connected GGM module (*p* < 5.9 × 10–7).

To further validate the accuracy of this method, we also examined connectedness of the following two subsets of the 88 genes known to be co-regulated: genes with RAREs^30^ and those associated with parvin-beta (*PARVB*) by GSEA. Significant connectedness was indeed found for both sets (*p* < 4.5 × 10−3, *p* < 1.71 × 10−9, respectively), suggesting that the method is sensitive. In contrast, as expected, three random gene sets did not show significant connectedness (Supplementary Table 3).

Taken together, these results support the hypothesis that MSLN interacts with *RARG* and *TNK2*, though other factors and feedback loops could not be ruled out. Indeed, the first order neighbors with strong edges to *MSLN*, *RARG*, and *TNK2*, such as *PITX1*, *KCNN4*, *ALS2CL*, and *TRAK1*, could also play roles in the MSLN-induced expression of *RARG* and *TNK2* (Fig. 4c).

## Discussion

The proposed histoepigenetic analysis method generates novel mechanistic hypotheses by detecting differentially expressed genes within minimally perturbed cancer cells in their tumor microenvironment and places them in a network context. By eliminating artifacts associated with physical separation of cells and their propagation in vitro, the method produces hypotheses that are testable in tractable models such as cell lines while ensuring relevance of the knowledge gained for human tumor biology in vivo. The method extends the previously developed EDec method by extending the deconvolution step with an iterative two-way clustering step to group tumor samples based on cancer cell-type-specific expression of a gene of interest. In the second stage, the gene is placed in network context. Using the example of *MSLN* in PDAC, we demonstrate how the method identifies potentially interacting genes and pathways associated with MSLN within the cancer cell in its native microenvironment. In contrast, most previous mechanistic studies of MSLN-associated pathways started with cell lines or mouse models. This general approach can be applied to patient tumor samples from other cancer types to study other genes of interest.

We demonstrate the utility of the method by elucidating interactions within the *MSLN* network in PDAC cancer cells. The analysis identified the *MSLN*-associated network module and interactions downstream of MSLN that could be validated in cell line models. Given that our results suggest that MSLN is necessary, but not sufficient for *RARG* and *TNK2* expression, future work is required to fully understand the precise mechanisms of how MSLN is acting upstream of these genes. The *MSLN* network module contains a number of candidate genes that may play a role in this mechanism.

Our findings complement current understanding of RARγ and retinoids as important players in PDAC biology and normal development. Because RARγ expression is reduced upon *MSLN* knockdown in cell line experiments, RARγ has the potential to act downstream of MSLN. RARγ acts in the cytoplasm and nucleus to repress or activate genes depending on the context, cofactors, and abundance of its retinoid ligands (reviewed in ref. ^31^). Specifically, RARγ regulates the balance of self-renewal and differentiation^31,32^. In the developing pancreas, retinoids affect ductal versus acinar differentiation^27^. Treatment of some pancreatic cancer cell lines with retinoids induces apoptosis, epithelial differentiation, cell cycle arrest, and reduces cell viability^33–35^. Some of these effects are mediated by RARγ as observed in the BxPC3 and Capan2 cell lines^33^, the same cell lines in which we showed reduction of RARγ upon *MSLN* knockdown. Conversely, cell lines with low RARγ appear resistant to this effect^33^. Moreover, patient PDAC tumors show reduced levels of retinoids compared to normal counterparts^36^. Thus, MSLN’s observed effect on pancreatic cancer cell proliferation in vitro^4^ may partly be mediated by RARγ and its ability to regulate the balance of self-renewal and differentiation depending on the concentrations of retinoids present.

Another important member of the MSLN network is AKT. There are conflicting reports in the literature regarding the ability of MSLN to affect the phosphorylation and activation of AKT in PDAC cell lines^5,7^, which is associated with worse prognosis in PDAC patients^37^. Similar to the effects of MSLN in PDAC cell lines^3–5^, activated AKT inhibits apoptosis, is involved in cell cycle progression, and mediates cell proliferation^22,38^. Our results expand upon the notion that MSLN may activate AKT, suggesting that this activation may be mediated through RARγ or ACK1. Moreover, because MSLN inhibition decreases phospho-AKT levels in other cancers with elevated MSLN levels^39^, our results may also be relevant for cancers other than PDAC.

The interactions within the *MSLN* network have significant implications for current PDAC drug discovery efforts targeting MSLN, RARγ, and their network neighborhood. Specifically, the limited benefit of targeting MSLN may be examined in the context of the tumor-specific state of the downstream mediators suggested by our study. Moreover, the pattern of interactions within the *MSLN* module has implications for utilizing retinoids and ACK1 inhibition as therapeutic strategies. Pilot clinical trials utilizing retinoids—most not selective for RARγ—have shown limited or no effects, with only some pancreatic cancer patients showing prolonged stable disease^40,41^. Preclinical trials showing that inhibition of ACK1 in PDAC cell lines has resulted in AKT suppression and subsequent decrease in cell proliferation, cell cycle arrest, and induction of apoptosis^22^. Our results suggest that these therapeutic targets should not be viewed in isolation but rather in relation to one another. In addition to the already tested targets, the *MSLN* network module includes new candidates that may play important roles. Finally, the suggested interactions within the *MSLN* network can be utilized to identify candidate biomarkers for stratification of patients or as targets for combination therapies.

## Materials and methods

### Processing of the TCGA PDAC dataset

Level 3 DNA methylation, RNA-seq (HiSeqV2), and clinical metadata datasets from the TCGA Pancreatic Cancer cohort (PAAD), not flagged during previous review^15^, were downloaded from UCSC Xena^42^. For the methylation dataset, probes with a missing beta value for any individual sample or falling on sex chromosomes were removed. Problematic 450 K probes^43^ were filtered using the mapping, typeINextBaseSwitch, snp5.common, extBase, and sub30.copy masks from http://zwdzwd.io/InfiniumAnnotation/current/hm450/hm450.hg38.manifest.tsv.gz and multi-mapping, cross-reactive, and probes containing common SNPs from https://github.com/sirselim/illumina450k_filtering. Probes associated (FDR < 0.01) with gender, radiation therapy history, history of diabetes, history of pancreatitis, smoking status, and history of targeted molecular therapy were determined by fitting an analysis of variance model using R’s aov function and removed. As were those associated with age and weight, determined by fitting linear models using R’s lm function.

### Deconvolution of TCGA datasets using EDec

The *EDec* R package was used to infer cell-type-specific methylation and gene expression profiles as previously described^17^. 500 informative probes were chosen by running the run_edec_stage_0 command with the “one.vs.rest” option and *p* value < 0.00001 for six selected reference groups using available methylation profiles from NCBI’s Gene Expression Omnibus^44^ (GEO) (Supplementary Table 4). Only probes present in the processed TCGA PDAC methylation dataset and with detection *p* < 0.01 across all reference profiles were retained. The appropriate number of cell types for deconvolution was determined with EDec’s estimate_stability function as previously described^17^ using reproducibility of stage 1 estimated matrices as the criterion. Estimated mean gene expression values and standard errors were used to construct bar plots using the *ggplot2* package in R.

### The first stage of histoepigenetic analysis: iterative computational deconvolution centered on a gene of interest

As illustrated in Fig. 2a, the matrix of cell proportions derived by stage 1 EDec deconvolution (yellow matrix in Fig. 2a) is used for iterative computational deconvolution to estimate sample-specific cancer-cell intrinsic expression of a gene of interest (arrows from the yellow matrix to the left and right in Fig. 2a). Specifically, the matrix of bulk gene expression profiles of all 150 TCGA samples were randomly split into two submatrices and independently input into stage 2 of EDec to estimate the cell-type-specific expression matrix for each group using the proportion matrix estimated from stage 1. For each sample, the linear combinations of its composition estimate with each of the two groups’ cell-type-specific expression profiles are calculated. The Euclidean distances were compared between each group’s combination and the sample’s bulk expression of *MSLN* and the sample was assigned to the group with shorter distance. The grouping and deconvolution steps were iterated until samples were no longer reassigned between groups. Three complete rounds of this iterative procedure were performed to test stability of final groupings. Groupings were formed on the consensus of these 3 runs.

### Simulating methylation and RNA-seq mixtures

Methylation datasets for four cell types (B-cell, Fibroblast, Capan2, and MIA Paca2) were downloaded from GEO (GSM861675, GSM1669664, GSM1670109, GSM2420490). Noisy profiles were generated for each dataset and then the immune, fibroblast, and one of two cancer profiles were mixed in random proportions as previously described^17^. Each cancer profile was used in fifty mixtures. These 100 simulated methylation mixtures were deconvoluted using EDec. The estimated proportions and methylation profiles were compared to the known true proportions and original methylation profiles using Pearson’s correlation. For simulated RNA-seq profiles, FASTQ files for four publicly available RNA-seq profiles (GSM1576391, GSM2100912, GSM3133134, GSM3430680) were downloaded from GEO, QC’d using FastQC, processed using Trim Galore, and aligned using RSEM. The RNA-seq profiles were multiplied by the same proportions as their matched methylation mixtures for all 100 mixtures and reads for each gene per mixture summed to create the mixture’s bulk profile. The iterative group fit method was used to group the RNA-seq mixtures around *MSLN* expression. Splitting was performed 20 times to check stability and all possible pairwise correlations of the final group members across these runs were compared. Groups were then deconvoluted separately.

### Comparison of differential expression methods

For the TCGA samples, *MSLN* high (*n* = 30) and low (*n* = 35) groups were determined by overlapping samples classified by both the bulk *MSLN* and the iterative group fit models. The deconvoluted cancer cell profiles for the groups were compared for differential expression using t-tests with the estimated means and standard errors of genes as previously described and with the same caveats^17^. Bulk differential expression between groups was done with raw (non-normalized) RSEM gene counts downloaded from NCI’s GDC Legacy Archive (https://portal.gdc.cancer.gov/legacy-archive/search/f) using the *DESeq2* R package^45^ with groups (*MSLN* high or low) with or without EDec-predicted cancer cell proportions as factors. All comparisons used a fold change ≥ 2 and false discovery rate (FDR) *q* value < 5%.

### Gene set enrichment analyses on cancer genes with higher expression in *MSLN* high group

The 33 genes with higher expression in the cancer cell profile of the *MSLN* high group were used for gene set enrichment analysis using the Molecular Signatures Database (MsigDB) web site version 6.3 (ref. ^46^). The top ten overlaps of these genes were computed against each of the C1–C6 gene set collections using an FDR *q* value < 0.05. The 689 genes that showed higher expression in the *MSLN* high group at a *p* value < 0.05 were compared to the gene list AKT_expression_UP from MsigDB. Enrichment for the 13 gene overlap with the 172 AKT genes was computed using a hypergeometric test.

### The second stage of histoepigenetic analysis: construction of the gene network

As illustrated in Fig. 2a, in the second stage of histoepigenetic analysis a network is constructed and within it a module is identified that contains the gene of interest. The network is constructed as a GGM. To select which GGM learner to use, two methods were evaluated over three criteria: (1) The ability to form highly connected modules of previously known interactors, (2) the lack of modules from randomly selected gene sets, and (3) directionality of edges (positive or negative) matching biological predictions. R packages *huge*^47^ (graphical lasso method) and *GeneNet*^48^ (empirical Bayes method) were used to build networks over log_2_ transformed RNA-seq values for all 150 TCGA tumor samples on the 367 differentially expressed genes and the proportions of each cell type per sample. Positive controls were a set of 7 RARG related genes and a set of 21 genes related to *PARVB* expression and negative controls were randomly selected gene sets of size 7, 21, and 88 (Supplementary Table 5).

The *huge* network was trimmed using the Stability Approach to Regularization Selection^49^ (StARS) method (λ = 0.125). *GeneNet* created two networks, one with all edges and one with the same number of edges present in the *huge* network. To eliminate confounding due to variation in cell type composition, a node representing cell proportion was added (arrow from the yellow matrix down in Fig. 2a). After network construction, this node and its adjacent edges were removed for the purpose of subsequent analyses.

The R package *CTD* was used to quantify the modularity of the positive and negative control gene sets (Supplementary Table 3). *CTD* quantifies the relative level of connectedness (i.e. modularity) of a subset of nodes in a network, giving highly connected subsets in the network stronger significance compared to sparsely connected node subsets.

To assess the directionality of the edge weights, two sets of edge weights were evaluated between: (1) Genes predicted to be differentially expressed in *MSLN* high cancer cells and *MSLN* and (2) 5 genes with RAREs in their regulatory regions^30^ and *RARG* (Supplementary Tables 3 and6). The *huge* network did not agree with the expected directionality, but was consistent with the evaluation of relative modularity. In contrast, both *GeneNet* networks showed more consistent directionality, but poor sensitivity of modularity. Because modularity was considered more important than directionality, we selected the *huge* network. Edges with weights exceeding the mean (µ = −0.00492) by at least one standard deviation (σ = 0.0297) were considered as being strong.

*CTD* was used to query the cancer, stromal, and immune specific dysregulated genes, as well as the 3-gene subset of *MSLN*, *RARG*, and *TNK2* for modularity. Enrichment of edges was computed using a hypergeometric test.

### Cell culture

PDAC cell lines were purchased from ATCC, authenticated by short tandem repeat profiling, and propagated for less than 6 months after authentication. *MSLN* overexpression stable cell lines of Panc1, Panc28, and Panc48, as well as *MSLN* shRNA knockdown cell lines of Capan2, CFPAC1, and PL45 were established and maintained as previously described^50^. ASPC1, BxPC3, and SU86.86 cells were cultured in RPMI 1640 (Gibco, Thermo Fisher Science, USA); CFPAC1, HPAF-2, MIA Paca2, Panc1, Panc28, Panc48, and PL45 cells were cultured in DMEM (Gibco, Thermo Fisher Science, USA); Capan2 cells were cultured in McCoy’s 5 A medium (Gibco, Thermo Fisher Science, USA). All cells were supplemented with 10% fetal bovine serum (Gibco, Thermo Fisher Science, USA) and cultured at 37 °C with a humidity of 90–95% and 5% CO_2._

### Real-time PCR

Total RNA for each cell line was extracted using TRIzol reagent (Invitrogen, Thermo Fisher Science, USA) according to the product’s manual. cDNA synthesis was performed with a reverse transcription-PCR kit (Bio-Rad) and real-time PCR was performed by standard procedures as previously described^51^. *RARG* and *MSLN* measurements were normalized to *GAPDH* in the PDAC cell panel and relative to controls in the overexpression and knockdown comparisons. Primer sequences (5′–3′):

Human *MSLN* forward CTATTCCTCAACCCAGATGCGT and reverse GCACATCAGCCTCGCTCA,

Human *RARG* forward CAAGTGCATCATCAAGATCGTG and reverse GATACGCAGCATCAGGATATCT,

Human *GAPDH* forward TCGACAGTCAGCCGCATCT and reverse CCGTTGACTCCGACCTTCA.

### Immunoblotting assay

Cell lysates were prepared using RIPA lysis buffer with protease and phosphatase inhibitor cocktail and were quantitated for protein abundance using the Pierce BCA Protein Assay Kit from Thermo Fisher Scientific. PVDF membranes were blocked in 5% nonfat dry milk/TBST solution and incubated at 1:1000 dilution with: MSLN mAb (D9R5G), RARγ1 mAb (D3A4), AKT Ab #9272, Phospho-AKT (Ser473) mAb (D9E), Phospho-AKT (Thr308) mAb #2965, and Phospho-ACK1 (Tyr284) Ab#3138 from Cell Signaling Technology and ACK1 antibody (A-11) from Santa Cruz Biotechnology. Anti-β-actin Ab (A2228) was at 1:2,000 dilution (Sigma-Aldrich). Immunodetection was performed using SuperSignal West Pico (34577) and Femto (34095) Chemiluminescent Substrate according to the manufacturer’s instructions (Thermo Fisher Scientifc, USA).

### Statistical analyses

Unless otherwise noted, all statistical analyses and bioinformatics were performed using the R programming language version 3.4.0 (https://www.R-project.org/). *P* values were adjusted for multiple comparisons using the Benjamini & Hochberg method. Survival analyses were done with the R package *survival*, including multivariate Cox regression using variables (Supplementary Table 7) with *p* values < 0.05 in univariate Cox regression. Real-time PCR results were representative of three independent experiments and compared with two-tailed unpaired Student’s *t*-test with Welch correction when sample variances were not equal as defined by the Brown–Forsythe test using GraphPad Prism Software Version 6 and 7.

## Supplementary information

Supplementary Information

Supplementary data

Supplementary data

Supplementary data

Supplementary data

## Data Availability

The expanded EDec methodology for grouping samples around the expression of a gene of interest and CTD methodology are available as R packages at: https://github.com/BRL-BCM/PDAC_decon and https://github.com/BRL-BCM/CTD.
