# Supplementary material for: Histoepigenetic analysis of the mesothelin network within pancreatic ductal adenocarcinoma cells reveals regulation of retinoic acid receptor gamma and AKT by mesothelin
Source: Oncogenesis. 2020 Jul 2;9(7):62. doi: 10.1038/s41389-020-00245-3 (PMC7332500; doi:10.1038/s41389-020-00245-3)
Supplement: Supplementary file 1 — Supplementary Information [file 41389_2020_245_MOESM1_ESM.doc]

**Supplementary Information**

**Supplementary Figures**

**
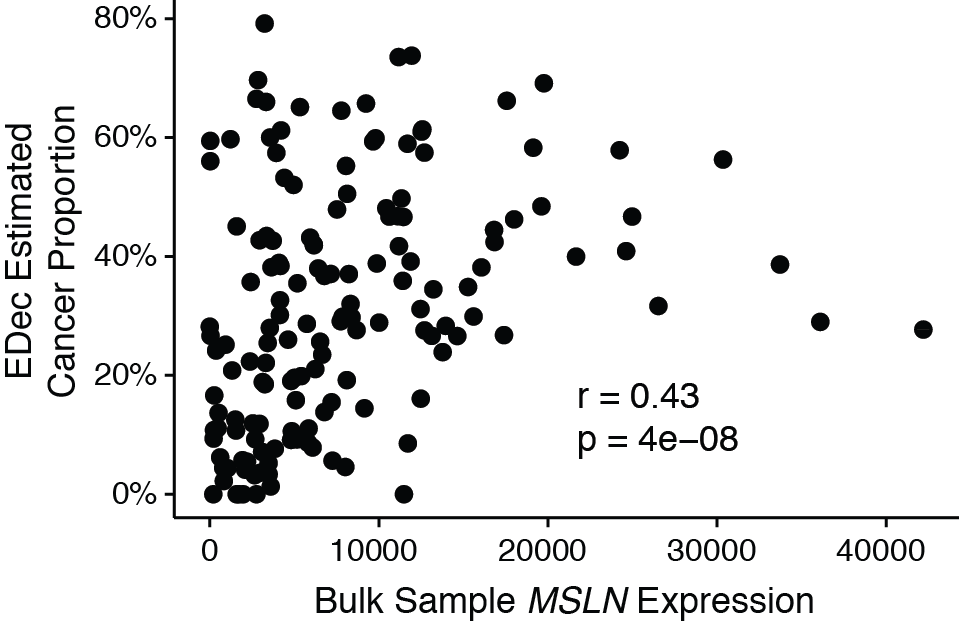
**

**Supplementary Figure 1**

Scatter plot showing Spearman’s correlation between each sample’s estimated cancer cell proportion with its bulk level of *MSLN* expression from the 150 tumors in the TCGA collection.

**
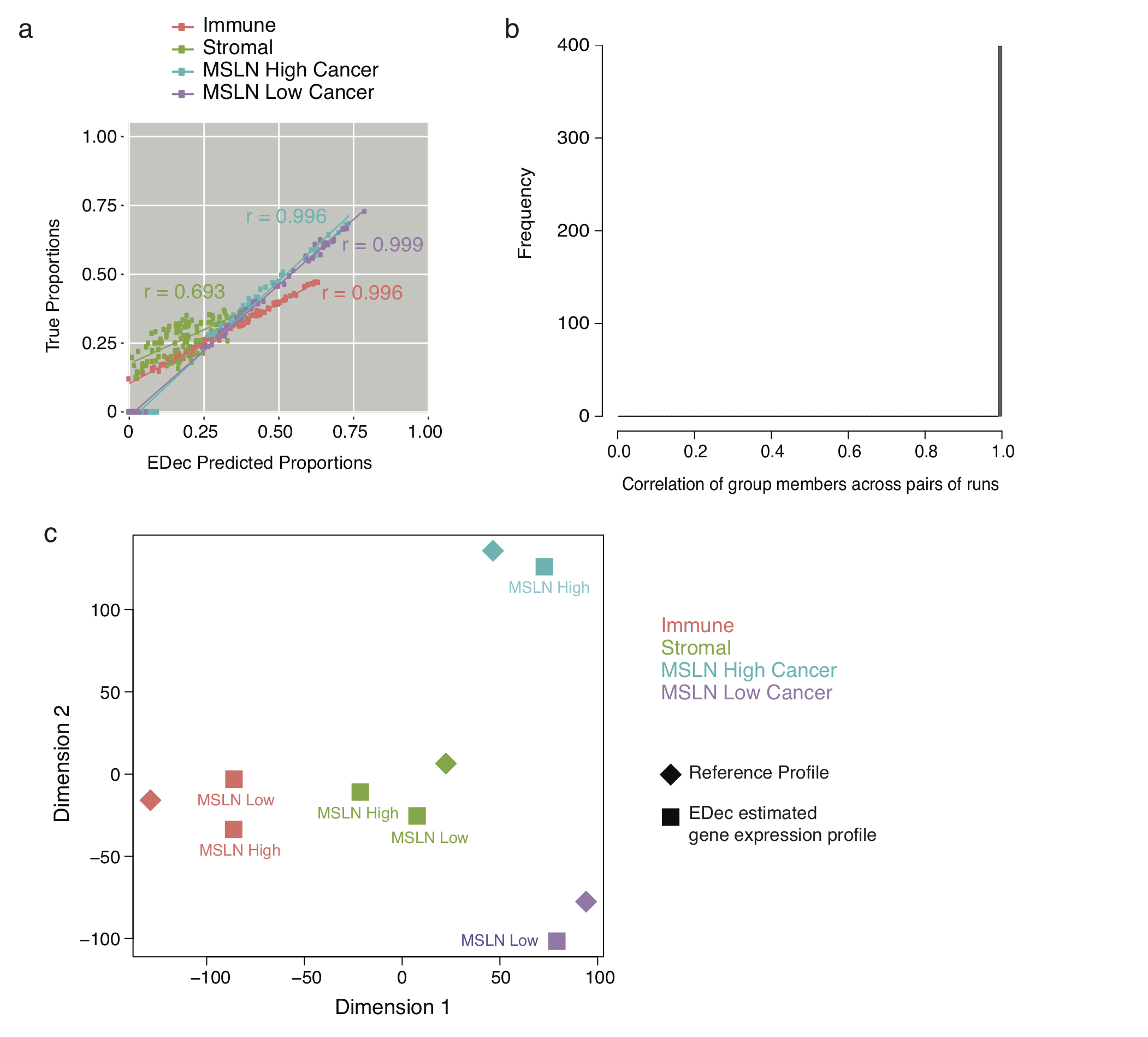
**

**Supplementary Figure 2**

Histoepigenetic analysis of the simulated dataset. **a.** Scatter plot showing Pearson correlations between known true proportions and estimated proportions for each of the 100 simulated mixtures for each of the 4 cell types. **b.** Histogram of correlations between group membership across all possible pairs of 20 runs of iterative deconvolution. **c**. T-distributed Stochastic Neighbor Embedding (t-SNE) plot showing clustering of deconvoluted cell-type specific profiles with references used to generate the simulated mixtures.

**
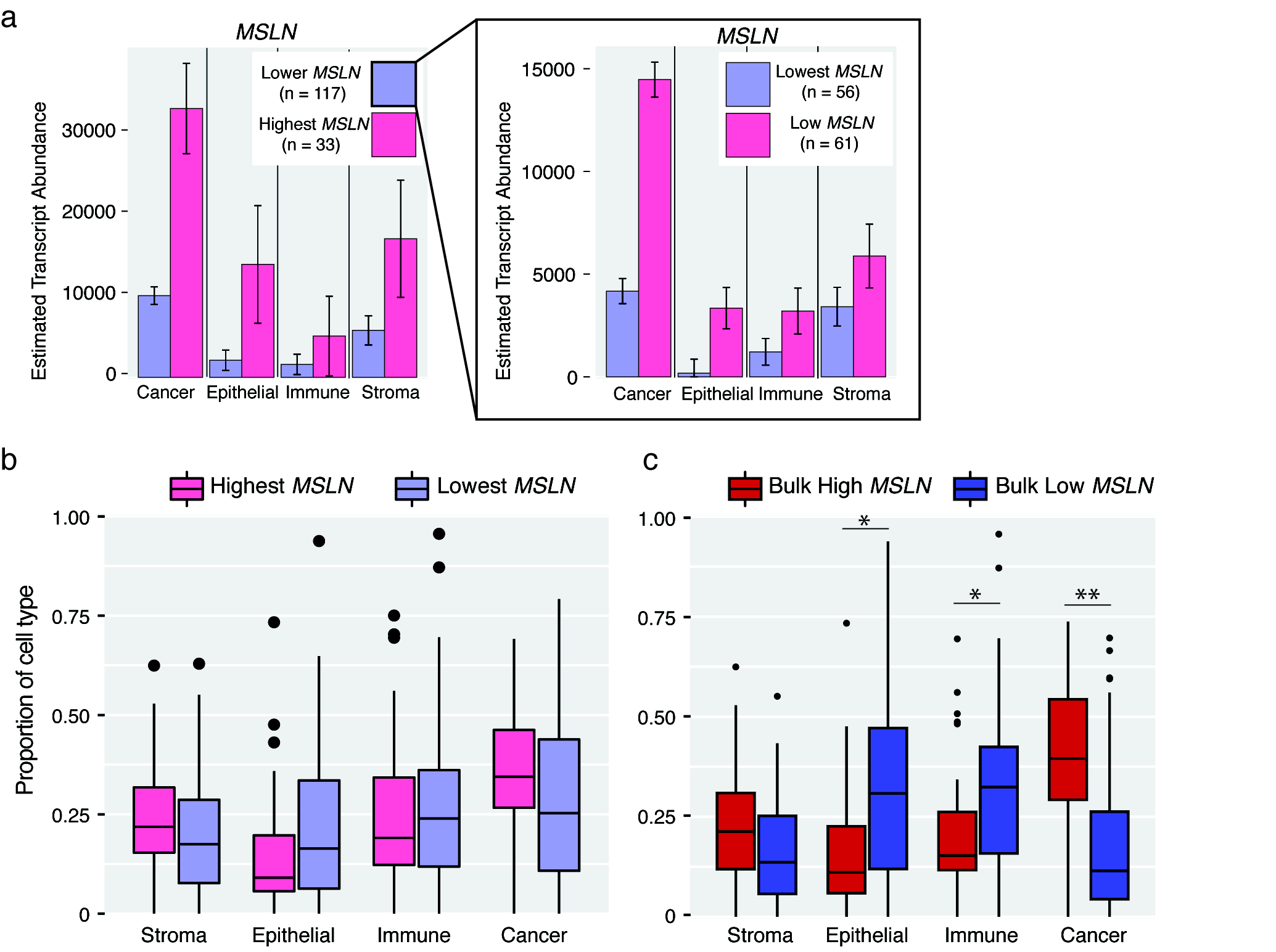
**

**Supplementary Figure 3**

Stage 1 (iterative deconvolution) of histoepigenetic analysis of PDAC profiles within the TCGA collection, centered on *MSLN* expression. **a.** Bar plot of mean *MSLN* expression, with standard error, in the four predicted cell types from the deconvolutions of *MSLN* high (light red) and lower (light blue) groups. Callout shows further split of the low group into two. **b.** Box plots showing estimated proportions of the 4 predicted cell types for the cancer cell intrinsic *MSLN* highest (light red) and lowest (light blue) groups. **c.** Box plots showing estimated proportions of the 4 predicted cell types for the bulk *MSLN* high (red) and low (blue) groups (* p < 0.05, ** p < 0.01).

**
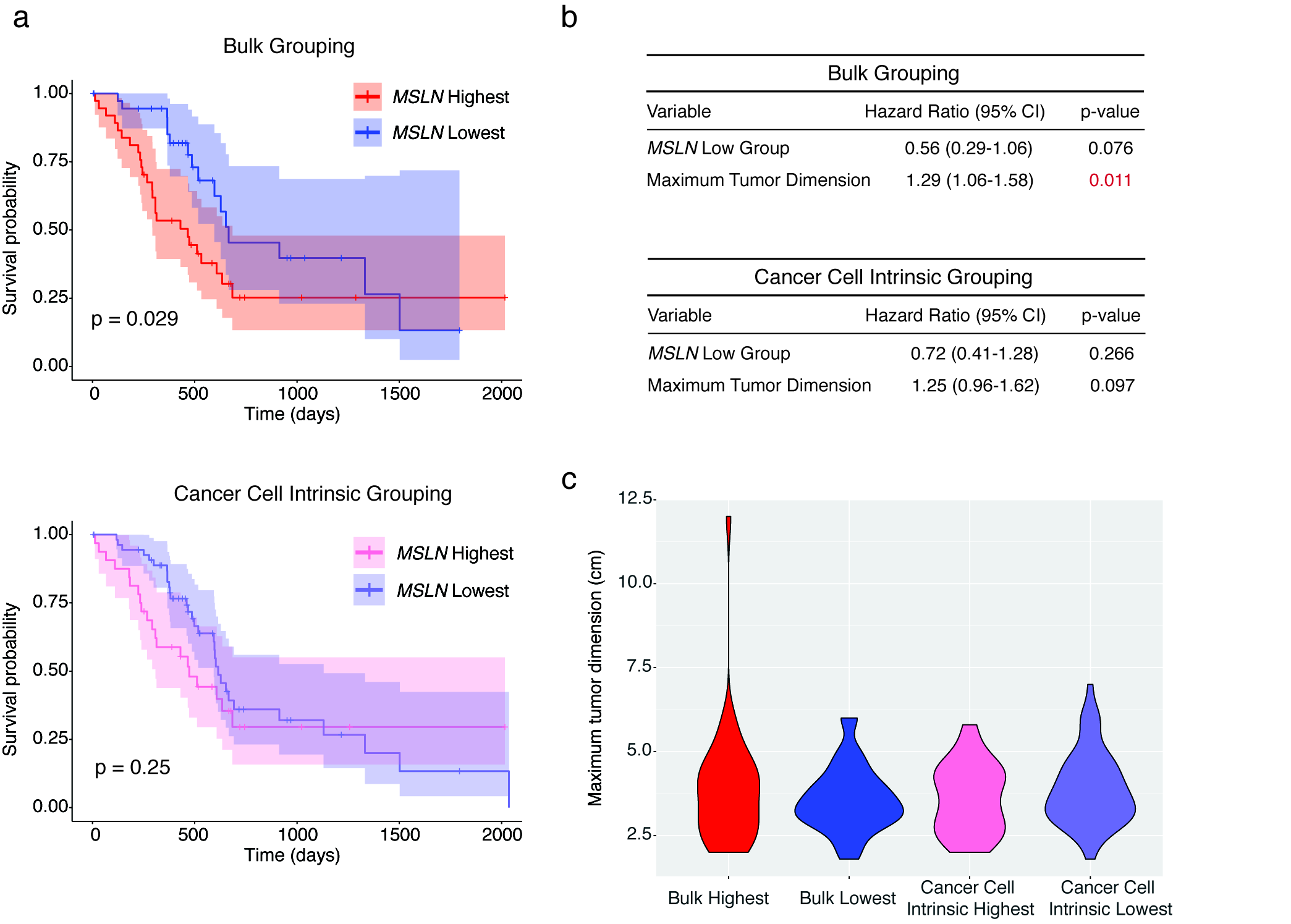
**

**Supplementary Figure 4**

Overall survival differences between grouping methods. **a.** Kaplan-Meier plots showing the differences in overall survival between *MSLN* high (red) and low (blue) groups, where the groups are defined either from bulk expression (top) or cancer-cell specific expression (bottom) with log-rank test p-values. **b.** Multivariate Cox regression analysis of time to death for groupings based on bulk (top) or cancer cell intrinsic (bottom) *MSLN* levels. **c.** Violin plots showing maximum tumor dimensions of samples in the bulk high (red), bulk low (blue), cancer cell intrinsic high (light red), and cancer cell intrinsic low (light blue) *MSLN* groups.

**Supplementary Tables**

**Supplementary Table 1 -** Differentially expressed cancer genes between *MSLN* high and low groups of tumors. Attached as separate .xls file.

**Supplementary Table 2** - Gene set enrichment results for various gene sets. Attached as separate .xls file.

| Modularity | **7 RARG**  **genes** | **21 PARVB**  **genes** | **7 random**  **genes** | **21 random**  **genes** | **88 random**  **genes** |
| --- | --- | --- | --- | --- | --- |
| Huge - auto select | 0.000527462 | 1.69E-09 | 1 | 1 | 1 |
| Huge - proportions included | 13.76 | 3.18E+01 | 1 | 1 | 1 |
| GeneNet - all edges | 1 | 1 | 1 | 1 | 1 |
| GeneNet - match Huge | 1 | 1 | 1 | 1 | 1 |
|  |  |  |  |  |  |
| Directionality (RARE genes) | ***KCNN4*** | ***PITX1*** | ***ALS2CL*** | ***TNK2*** | ***CAMK2N1*** |
| Huge - auto select | (-) | (-) | (-) | (-) | (-) |
| Huge - proportions included | (-) | (-) | (-) | (-) | (-) |
| GeneNet - all edges | (+) | (+) | (+) | (+) | (+) |
| GeneNet - match Huge | (+) | N/A | N/A | N/A | (+) |

**Supplementary Table 3**

Three network construction models were tested for their ability to detect modules and the directionality of correlations. (***Top***) CTD was used to test the modularity of: a set of 7 genes related to RARG, 21 genes related to PARVB, and 3 sets of random genes (p-values reported). (***Bottom***) The direction of the correlation between 5 genes with known RAREs and *RARG* if edges were present. Edges were expected to show a positive correlation.

**Supplementary Table 4-** List of GEO references used for EDec stage 0. Attached as separate .xls file.

**Supplementary Table 5 -** Random subsets used to test networks. Attached as separate .xls file.

| Directionality (Upregulated 32) | Correct | Incorrect | No Edge |
| --- | --- | --- | --- |
| Huge - auto select | 0 | 13 | 19 |
| GeneNet - all edges | 22 | 10 | 0 |
| GeneNet - match Huge | 11 | 2 | 19 |
| Directionality (Down regulated 55) | Correct | Incorrect | No Edge |
| Huge - auto select | 0 | 7 | 48 |
| GeneNet - all edges | 29 | 26 | 0 |
| GeneNet - match Huge | 7 | 3 | 45 |

**Supplementary Table 6 -** Directionality of edges connected to *MSLN* (87 cancer cell genes).

| **Variable Tested** |
| --- |
| *MSLN* group (High or Low) |
| Proportion of estimated immune cells |
| Proportion of estimated stromal cells |
| Proportion of estimated normal epithelial cells |
| Proportion of estimated cancer cells |
| Age at diagnosis |
| Maximum tumor dimension |
| Gender |
| Histologic grade of neoplasm |
| Race demographic |
| Tumor stage at diagnosis |
| Clinical M-stage |
| Clinical T-stage |
| Clinical N-stage |

**Supplementary Table 7 –** Covariates tested individually in univariate Cox analyses.
